# Introduction of medical assistants in France: an innovative approach to healthcare access?

**DOI:** 10.1093/eurpub/ckac131.306

**Published:** 2022-10-25

**Authors:** O Guyonvarch, Y Bourgueil, F Dugué

**Affiliations:** 1 Faculty of Medicine, Sorbonne University, Paris, France; 2 Health Chair, Sciences Po, Paris, France; 3 Center for Primary Care and Public Health, University of Lausanne, Lausanne, Switzerland; 4 Directorate of Public Health, Municipality of Paris, Paris, France

## Abstract

Epidemic transition, sustained costs and health workforce shortage challenges have led numerous countries to strengthen primary care (PC) and implement new models of care. Faced with declining numbers of general practitioners (GPs), France has introduced medical assistants (MAs) in 2019 to guarantee access to care and maintain workforces in deprived areas. Trained to perform administrative and clinical tasks delegated by a physician, MAs are expected to optimize medical time and improve working conditions in practices. How does French model of MAs impact quality and productivity in GPs’ practices and articulate with other policies? We conducted a qualitative case study in 6 pilot practices to explore the effects of MAs’ work (interviews with 12 GPs, 6 MAs, collection of tasks performed by 6 MAs), complemented with views from public policy makers and health professional unions (9 interviews). MA was defined as a function centered on physicians’ needs, accessible both to administrative staff and nursing professions. MAs with a clinical profile performed a wider range of tasks, were more prone to perform clinical tasks and build developed interactions with patients, and seemed better fitted for chronic disease care management. Recruitment of MAs by physicians is supported with grants that decrease yearly while practice productivity is expected to rise. In general, a gain of efficiency in daily workload enabled GPs to slightly increase their productivity. However, for most GPs, it primarily helped them to maintain high workload without burning out. Although MAs with clinical background seem better suited for patients’ needs, recent figures have shown that more than half of MAs employed are former secretaries. If in-person secretaries could be endorsed with further administrative duties, MAs could hold a more clinical role in PC teams including physicians and allied health professionals. Other aspects than productivity must be taken into account in a support policy.

**Key messages:**

• Regardless of productivity objectives to attain, hiring MAs can relieve physicians’ workload and stress, preventing them from burning out and guarantee access to care in deprived areas.

• MA’s clinical profile could have a stronger impact on public health issues such as chronic disease care management.

